# Interdigital pilonidal cyst and its clinical and ultrasonographic aspects

**DOI:** 10.1016/j.jdcr.2024.10.033

**Published:** 2024-11-26

**Authors:** Giovana El Khouri Bechara, Carolina Scaff Haddad Bartos, Priscilla Tashiro Forster, Rosana Lazzarini

**Affiliations:** Department of Dermatology, Irmandade da Santa Casa de Misericordia de São Paulo, São Paulo, Brazil

**Keywords:** interdigital pilonidal cyst, occupational dermatology, ultrasound diagnosis

## Introduction

Interdigital pilonidal cyst is a rare occupational condition, also known as “barber's disease,” affecting professionals such as barbers, hairdressers, and animal groomers.^1^ It is caused by the penetration of hair fragments into the interdigital space after prolonged exposure.^2^ Hygiene and protective measures can assist in preventing this condition.^1^ We present a case of interdigital pilonidal cyst treated at the Dermatology Clinic in São Paulo.

## Case report

**Fig 1:**
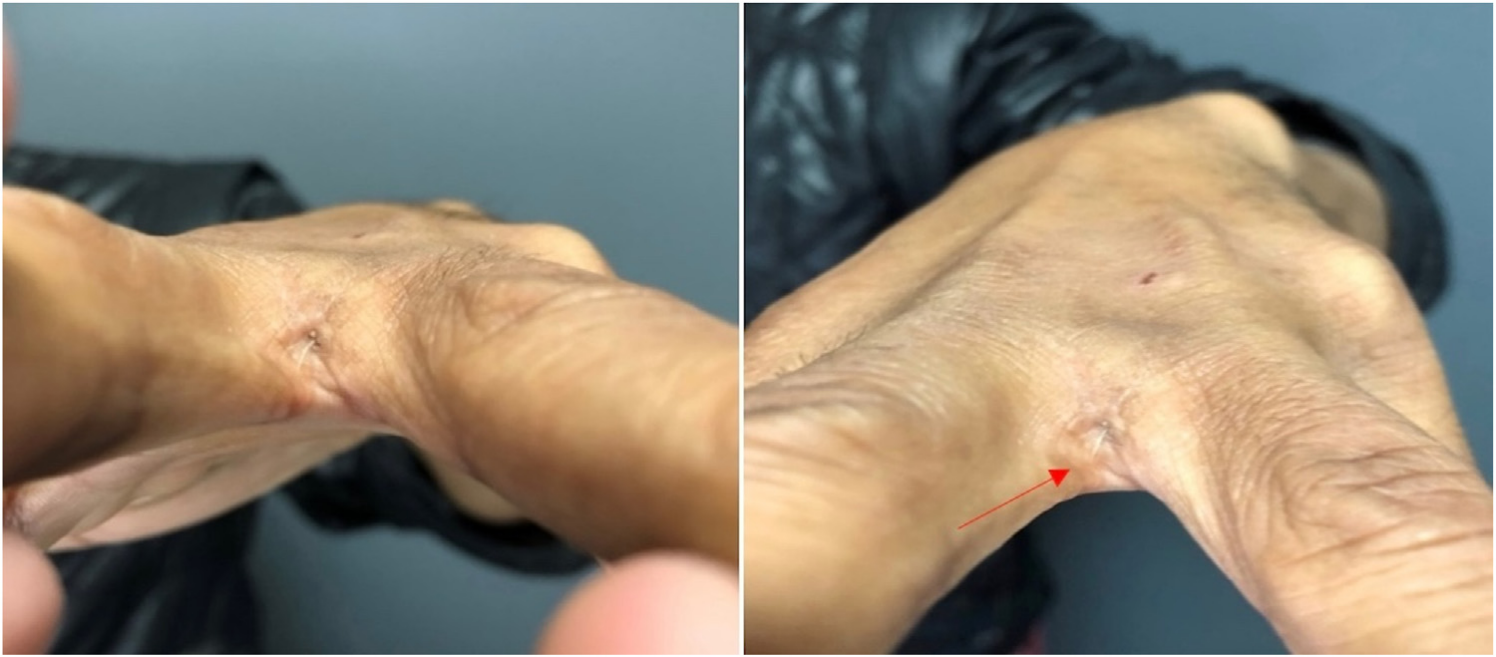
An orifice in the interdigital region between the third and fourth digits of the left hand, from which a hair shaft is observed emerging (*red arrow*).

**Fig 2:**
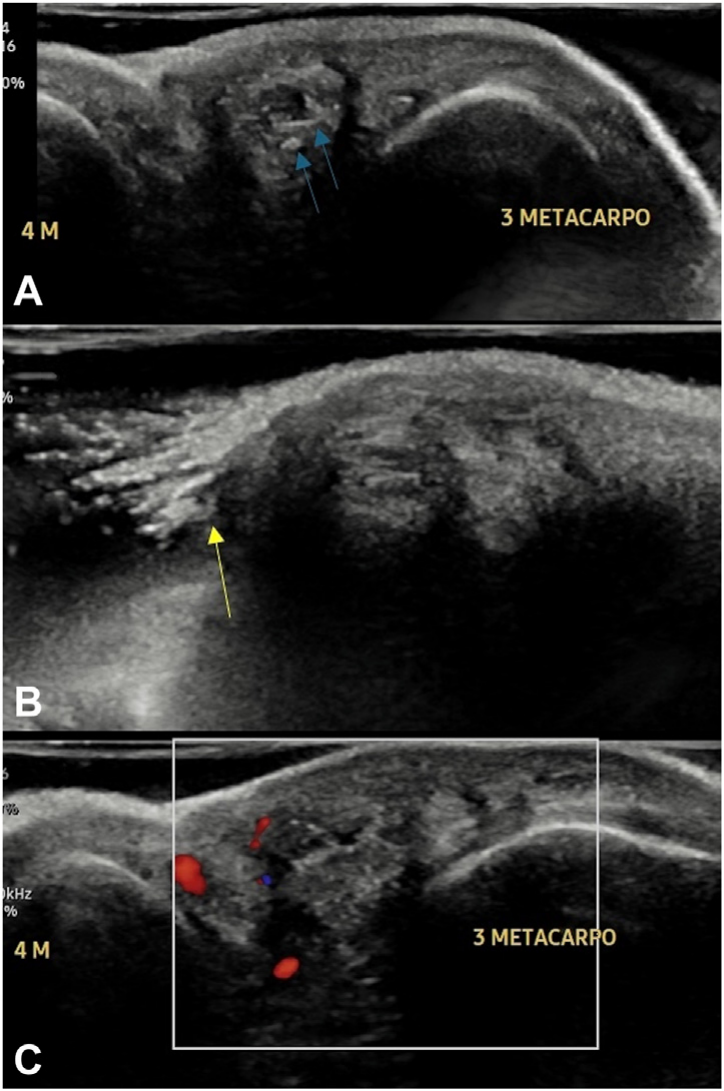
**A,** Ultrasound image showing a cross-section between the third and fourth left metacarpals, demonstrating a heterogeneous nodular formation with interspersed hyperechoic linear images (*blue arrows*) in the dermis and subcutaneous tissue; **B,** Longitudinal section showing the continuity of hyperechoic images through an area of discontinuity in the interdigital epidermis (corresponding to hair follicles), indicated by the *yellow arrow*. **C,** Doppler study of the lesion reveals predominantly peripheral vascularization.

A 45-year-old man, working as a groomer in a pet shop for 18 years, was referred by the primary healthcare unit due to a lesion in the interdigital region between the third and fourth left digits 12 years ago. He reported a similar lesion appearing 3 years ago in the same location on the contralateral hand. On dermatologic examination, the patient presented with a dermatosis between the third and fourth left digits, characterized by a 1 cm diameter papule with a central orifice from which a hair shaft was protruding (Fig 1). Ultrasonographic examination (Samsung HM70 EVO with LA-31-16AD transducer) revealed a nodular formation in the dermis and hypodermis, heterogeneous, predominantly hypoechoic, with linear hyperechoic images interspersed, extending to a tiny epidermal discontinuity (corresponding to the hair shaft), located between the third and fourth metacarpals on the left side. Doppler study showed predominantly peripheral vascularization (Fig 2). Histopathologic examination showed a follicular cyst with foreign body giant cell reaction. Surgical treatment was opted for, and the lesion was successfully excised without complications.

## Discussion

The term “pilonidal” is derived from Latin terms - *pilus* and *nidus*, meaning “nest of hairs.”^2^ The classic pilonidal cyst appears in areas of hair follicles and is rarely found in hairless areas, such as the interdigital region. When present in these locations, it is related to occupational and acquired dermatoses.^2^ The microscopic absence of the cyst wall and true epithelium in the excised tissue is the most important evidence that the lesion does not develop from remnants of congenital tissue and is thus described as an acquired disease.^3^

Reports of pilonidal cysts in the interdigital region of the hands are rare, with the first description made by Allington and Templeton in 1942. Also known as “barber's disease,” it can affect other professions such as hairdressers, pet shop groomers, cow milkers, and sheep shearers.^1^

The hair of men, when cut, is often sharp, thick, and straight, capable of piercing the thin epidermis of the interdigital region of the professional.^1^^,^^2^ After implantation of the hair, a foreign body reaction associated with chronic inflammation begins, with granuloma formation and subsequent formation of a cyst filled with hair shafts.^1^, ^2^, ^3^ In general, the female hair is characterized by being long, fine, soft, and flexible, with the tips being more predisposed to bending before penetrating the skin. This is the reason why most described cases occur in barbers.^2^ The condition is generally asymptomatic, but over time, secondary infection may occur with purulent drainage and possible progression to abscess, cellulitis, and even osteomyelitis.^3^

The diagnosis is clinical and epidemiologic, confirmed by histopathologic examination showing the presence of foreign body granuloma.^2^^,^^3^ Recently, the introduction of imaging tests such as ultrasound in dermatology has helped in diagnosis, as in the case described here. Ultrasonographic imaging demonstrates the presence of the hair shaft, contributing to the etiologic clarification of the entity. Although it is an examination that requires accurate training, its low cost makes it important in managing cases.^4^

The interdigital pilonidal cyst should be considered alongside other cystic skin lesions, such as epidermoid cysts, subcutaneous abscesses, and foreign body granulomas. These conditions share clinical features, however they can have differing ultrasonographic findings. Epidermoid cysts, for example, appear as well-defined, oval or round structures in the superficial subcutaneous tissue, often showing posterior acoustic enhancement and internal echogenicity due to keratin and cholesterol. Subcutaneous abscesses typically present with thickened walls and increased peripheral vascularity, while foreign body granulomas often have thick, avascular walls with echogenic material. Ultrasonography can aid in accurate diagnosis and appropriate clinical management of these entities.^5^

Total excision of the sinus tissue is considered the most effective treatment method, and prevention is the most efficient way to avoid the disease and its recurrence, as performed in this case.^1^, ^2^, ^3^, ^4^ Prevention involves proper hygiene routine, including hand cleaning and drying, glove use, and removal of any hairs that may penetrate the skin during work.^1^, ^2^, ^3^

## Conflicts of interest

None disclosed.
